# Icariin modulates osteogenic and adipogenic differentiation in ADSCs via the Hippo-YAP/TAZ pathway: a novel therapeutic strategy for osteoporosis

**DOI:** 10.3389/fphar.2024.1510561

**Published:** 2025-01-13

**Authors:** Shaozi Lin, Zuyu Meng, Mei Wang, Zixuan Ye, Mengsha Long, Yiyao Zhang, Fang Liu, Hongling Chen, Menghan Li, Jiajia Qin, Haiquan Liu

**Affiliations:** ^1^ School of Traditional Chinese Medicine, Jinan University, Guangzhou, China; ^2^ Huizhou Hospital, Guangzhou University of Traditional Chinese Medicine, Guangzhou, China

**Keywords:** Icariin, adipose-derived stem cells, HIPPO-YAP/TAZ, osteogenic, adipogenesis

## Abstract

**Background:**

Adipose-derived stem cell (ADSC) transplantation presents a promising approach for osteoporosis (OP) treatment. However, the therapeutic efficacy of ADSCs is hindered by low post-transplantation survival rates and limited capacities for adhesion, migration, and differentiation. Icariin (ICA), the primary active compound of Epimedium, has been shown to promote cell proliferation and induce osteogenic differentiation; however, its specific effects on ADSC osteogenesis and the mechanisms by which ICA enhances osteoporosis treatment through cell transplantation remain inadequately understood.

**Purpose:**

This study investigates the effects of different concentrations of ICA on the osteogenic and adipogenic differentiation of rat ADSCs, aiming to elucidate the underlying mechanisms. ADSCs were isolated from female SPF-grade SD rats, with surface markers identified through flow cytometry. Osteogenic and adipogenic differentiation were assessed using Alizarin Red and Oil Red O staining, respectively. Third-generation ADSCs were divided into five groups: control, resveratrol (100 μmol/L), and four ICA treatment groups (1, 10, 50, and 100 μmol/L). Western blotting was performed to analyze the expression of factors associated with the Hippo-YAP/TAZ signaling pathway and the adipogenic marker PPARγ. Additionally, ADSCs were labeled with lentiviruses carrying enhanced green fluorescent protein (EGFP) and 5-bromo-2-deoxyuridine (BrdU) to assess their *in vivo* distribution, survival, proliferation, and differentiation of ADSCs post-ICA intervention.

**Results:**

*In vitro*, ICA significantly inhibited the Hippo pathway, reducing YAP and TAZ phosphorylation and enhancing their transcriptional activity, while simultaneously suppressing PPARγ. This promoted osteogenesis and inhibited adipogenesis in ADSCs. *In vivo*, ICA-treated ADSCs demonstrated effective distribution, survival, and osteogenic differentiation following subcutaneous injection into allogeneic rats.

**Conclusion:**

Our study demonstrates that ICA significantly enhances the osteogenic differentiation of ADSCs while inhibiting adipogenesis, providing novel insights and therapeutic strategies for osteoporosis and related conditions.

## 1 Introduction

Osteoporosis (OP) is a systemic bone disease characterized by decreased bone mass, disrupted bone microarchitecture, and increased bone fragility (Ramchand and Leder, 2024). The primary causes of OP include reduced bone formation and increased bone resorption, leading to an imbalance in bone remodeling (Zhou et al., 2019). Current treatment options primarily focus on promoting bone formation or inhibiting bone resorption through pharmacological agents. However, these treatments do not reverse bone loss and are often associated with adverse effects, such as thrombosis and stroke. Thus, there is an urgent need for more effective therapeutic strategies that minimize side effects.

Mesenchymal stem cells (MSCs), the progenitors of both osteoblasts and adipocytes, are central to maintaining the balance between osteogenesis and adipogenesis, which is crucial for skeletal health (Rosen and Klibanski, 2009). A shift in MSC differentiation towards adipogenesis, resulting in increased adipocyte formation, has been implicated in the pathogenesis of OP (Kelly et al., 2013; Maurin et al., 2002). With advancing age, bone marrow adipocyte numbers increase, and elevated adipocyte levels in the bone marrow are often associated with decreased bone density (Bredella et al., 2009; Scheller et al., 2015). Furthermore, MSC differentiation into either osteocytes or adipocytes plays a critical role in various pathological conditions, including obesity and type 2 diabetes mellitus (T2DM) (Chen et al., 2016; Pino et al., 2012).

Adipose-derived stem cells (ADSCs) have emerged as a promising cell therapy for OP, demonstrating therapeutic potential comparable to that of bone mesenchymal stem cells (BMSCs) in rat models of ovariectomy-induced osteoporosis (Cho et al., 2012; Uri et al., 2018; Ye et al., 2014). ADSCs are abundant, easily obtainable, and associated with fewer donor-site complications, making them ideal candidates for regenerative therapies (Ciuffi et al., 2017). Previous studies indicate that in osteoporotic rats, BMSCs tend to differentiate into adipocytes, thereby diminishing their osteogenic potential. Therefore, inhibiting the adipogenic differentiation of ADSCs could enhance their osteogenic capacity.

The Hippo-YAP/TAZ signaling pathway plays a pivotal role in regulating stem cell fate, tissue homeostasis, and regeneration. By modulating the activity of YAP/TAZ, this pathway influences both osteogenic and adipogenic differentiation. Targeting the Hippo-YAP/TAZ pathway in ADSCs presents a promising approach to enhancing osteogenesis while concurrently suppressing adipogenesis, thus offering a novel strategy for the treatment of OP.

Icariin (ICA), a key bioactive compound derived from Epimedium, has been recognized for its use in traditional Chinese medicine for osteoporosis treatment. Research has shown that ICA downregulates peroxisome proliferator-activated receptor gamma (PPARγ) expression, promoting osteogenic differentiation and inhibiting adipogenic differentiation in osteoporotic rat BMSCs (Liu et al., 2017).

In this study, we investigated the effects of varying concentrations of ICA on the osteogenic and adipogenic differentiation of rat ADSCs and explored the underlying molecular mechanisms. ADSCs were treated with different concentrations of ICA, and the effects on key differentiation markers were assessed. Furthermore, ICA-treated ADSCs were transplanted into rats to evaluate their *in vivo* distribution, survival, and differentiation through immunofluorescence and immunohistochemistry techniques.

## 2 Methods

### 2.1 Animals

Five healthy, 4-week-old, SPF-grade nulliparous female SD rats were utilized for the preparation of ADSCs. The rats were sourced from the Experimental Animal Center of Guangzhou University of Chinese Medicine (License No: SCXK (Yue) 2016-0041) and housed in the SPF-grade animal facility at Jinan University.

### 2.2 Reagents

Icariin (molecular formula: C_33_H_40_O_15_; molecular weight: 676.7 g/mol; purity ≥98.0%; #110737-201516) and Resveratrol (molecular formula: C_14_H_12_O_3_; molecular weight: 228.243 g/mol; purity ≥98.0%; #111535-201703) were purchased from the China National Institutes for Food and Drug Control (Beijing, China).

### 2.3 Grouping

Resveratrol (RES) was utilized as a positive control, as it preferentially induces osteogenic differentiation in both bone marrow and adipose-derived stem cells (Bäckesjö et al., 2006; Erdman et al., 2012). Based on prior research and relevant literature (Yan et al., 2024a; Yan et al., 2024b), Icariin was set at a baseline concentration of 1 μmol/L, with additional groups at 10, 50, and 100 times this concentration. Resveratrol was used at 100 μmol/L, matching the highest Icariin concentration. Adipose-derived stem cells were divided into six groups: control group, resveratrol group (100 μmol/L), and Icariin groups (1, 10, 50, 100 μmol/L).

### 2.4 Isolation and culture of ADSCs

Adipose tissue was isolated from the inguinal and dorsal subcutaneous fat pads of the rats and thoroughly washed with sterile PBS to remove residual debris. The tissue was minced and digested at 37°C for 1 hour in 0.075% type I collagenase (#SCR103, Sigma) in α-MEM medium without FBS, with constant stirring. The digested cells were filtered and centrifuged at 300 g for 8 min. The cell pellet was washed three times in culture medium, resuspended in α-MEM medium supplemented with 10% fetal bovine serum, and cultured at 37°C in a 5% CO_2_ atmosphere. After 3–5 days, adherent cells were retained, and non-adherent cells were discarded. ADSCs were passaged three times before use in experiments, with cells utilized from passages no greater than P6.

### 2.5 Flow cytometry

Cells were digested with trypsin protease and resuspended in PBS at a concentration of 1 × 10^6^cells/100 μL. Cells were incubated with PE Anti-CD34 antibody [ICO-115] (#ab187284, Abcam) and FITC Anti-CD90/Thy1 antibody [EPR28145-53] (#ab307736, Abcam) at 4°C in the dark for 30 min. Stained cells were analyzed using a FACSCalibur™ flow cytometer (BD Biosciences), and data were processed using FlowJo 10 software. Each experiment was repeated three times.

### 2.6 Osteogenic and adipogenic differentiation assays of ADSCs

Third-generation ADSCs were divided into osteogenic and adipogenic induction groups and seeded into 6-well plates at a density of 1 × 10^5^ cells/well. Upon reaching 80%–90% confluence, differentiation was assessed on days 7, 14, and 21 using Alizarin Red staining for osteogenesis and Oil Red O staining for adipogenesis. The osteogenic group was cultured with the Osteogenesis Differentiation Kit (#A1007201, Gibco), while the adipogenic group utilized the Adipogenesis Differentiation Kit (#A1007001, Gibco). Media were changed every 3–4 days, and staining was observed under a microscope.

### 2.7 Western blot analysis

Following treatment, ADSCs were washed three times with PBS, and total proteins were extracted using RIPA lysis buffer containing protease inhibitors, phosphatase inhibitors, and 0.1 M PMSF. The lysates were centrifuged at 12,000 g at 4°C for 20 min, and total protein was quantified using the BCA method. Samples were separated by SDS-PAGE and transferred onto PVDF membranes. Membranes were blocked in 5% skim milk for 1 h, incubated with primary antibodies (1:1000) overnight at 4°C, and then washed with TBST three times. Secondary antibodies were incubated for 1 h at room temperature. Protein expression was detected using an ECL detection system (Bio-Rad). The antibodies used were YAP (#4912), p-YAP (#4911), TAZ (#83669), p-TAZ (#13008), GAPDH (#2118) from Cell Signaling Technology (Boston, MA, United States), and PPARγ (#ab19481-200) from Abcam (Cambridge, United Kingdom).

### 2.8 Lentiviral vector packaging and transduction

Lentiviral particles were generated using plasmids encoding the target gene along with helper plasmids (pGag/Pol, pRev, pVSV-G). The plasmids were purified using endotoxin-free methods and co-transfected into 293T cells using Lipofectamine™ 2000. Six hours after transfection, the medium was replaced, and cells were cultured for 72 h. The supernatant containing lentiviral particles was collected and concentrated. Viral titers were confirmed to exceed 1 × 10^8^ TU/mL by infecting 293T cells. ADSCs were transduced at various multiplicities of infection (MOI) to determine optimal infection efficiency, and cell status was observed using a fluorescence microscope.

### 2.9 Cell injection

Fifty-four 3-month-old rats were divided into six groups: control, resveratrol (100 μmol/L), and four different concentrations of ICA (1, 10, 50, 100 μmol/L). Labeled ADSCs (EGFP or BrdU) were injected subcutaneously into the rats. At 10, 14, and 21 days post-injection, five rats from each group were randomly selected for analysis. Tissue samples from the injection sites were collected for immunofluorescence and immunohistochemical analyses to evaluate cell survival, distribution, and proliferation.

### 2.10 Immunohistochemical detection of proliferation, osteogenic, and adipogenic markers (BrdU, ALP, Stro-1, PGC-1α, PPARγ)

Tissues were fixed in paraformaldehyde, permeabilized with Triton X-100, and blocked with goat serum. After overnight incubation with primary antibodies, the tissues were washed and incubated with secondary antibodies. DAB solution was used for staining, and observations were made under a microscope. Staining was terminated with distilled water, and coverslips were applied. Three random samples from each tissue were selected for analysis the IOD/area ratio using ImageJ software (Di et al., 2024; Zhang et al., 2023). The antibodies used were BrdU (#ab6326), ALP (#ab108337), Stro-1 (#ab102969), PGC-1α (#ab54481), and PPARγ (#ab19481-200) from Abcam (Cambridge, United Kingdom).

### 2.11 Statistical analyses

Statistical analyses were performed using SPSS 13.0 (IBM SPSS, Chicago, United States). The statistical comparisons between the groups were determined using one-way ANOVA, followed by Dunnett’s test, and graphs were generated using GraphPad Prism 7.0 (San Diego, CA, United States). Data are expressed as the mean ± SD of three independent experiments. A *p*-value <0.05 was considered statistically significant.

## 3 Results

### 3.1 Characterization and identification of ADSCs

**FIGURE 1:**
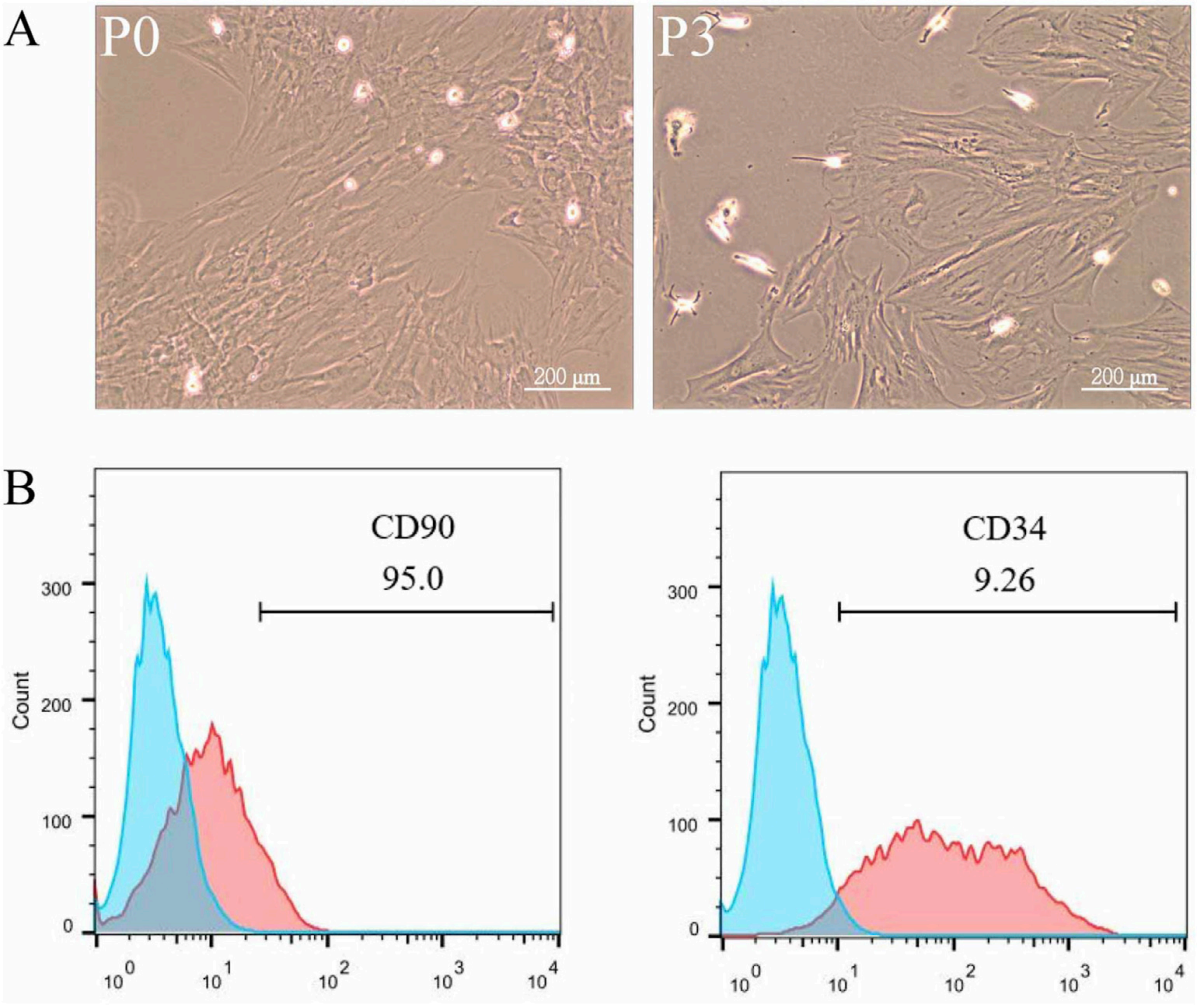
Characterization and Identification of ADSCs. Adipose tissue from rat fat pads was digested in collagenase in α-MEM without FBS for 1 h, filtered, centrifuged, washed, and resuspended in α-MEM with fetal bovine serum. ADSCs were passaged three times, being used up to P6. To analyze surface markers, cells were incubated with PE Anti-CD34 and FITC Anti-CD90 antibodies for 30 min, followed by flow cytometry and data analysis with FlowJo 10. The data were obtained from multiple analyses (n = 3). **(A)** Passage 1 ADSCs (left) and Passage 3 ADSCs (right) observed under ×100 magnification. **(B)** Flow cytometry analysis of ADSC surface markers CD90 and CD34.

Mononuclear cells isolated from rat adipose tissue were cultured and passaged in Petri dishes, displaying a fibroblast-like morphology with elongated, spindle-shaped cells (Figure 1A). Flow cytometry was employed to analyze the expression of cell surface markers, revealing a strong positive expression of the mesenchymal stem cell marker CD90 and a weak positive expression of the hematopoietic stem cell marker CD34, confirming that the isolated cells were adipose-derived mesenchymal stem cells (Figure 1B).

### 3.2 Differentiation ability of ADSCs

**FIGURE 2:**
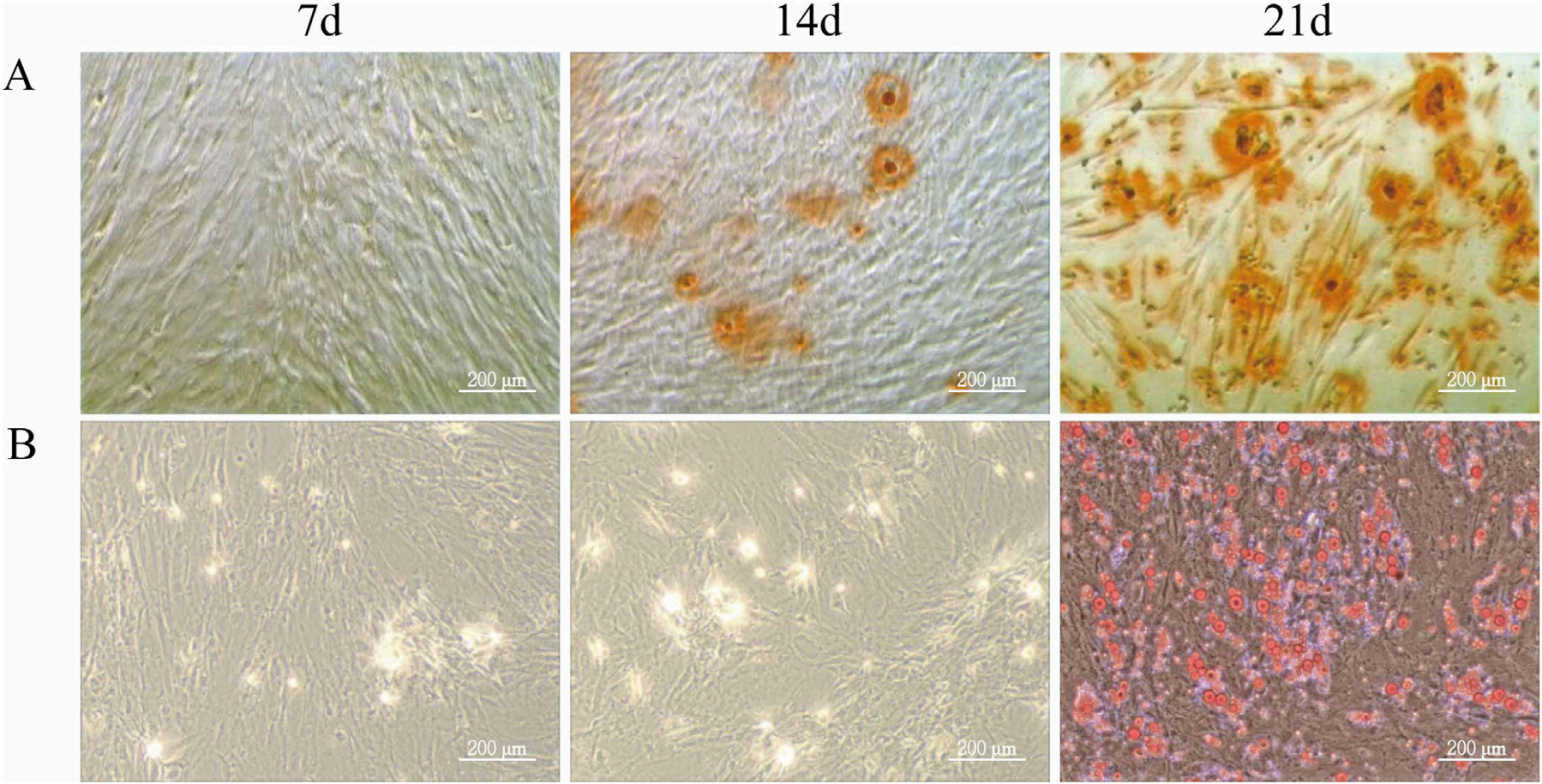
Differentiation Ability of ADSCs. Third-generation ADSCs were cultured in 6-well plates and induced for osteogenesis or adipogenesis. Differentiation was assessed on days 7, 14, and 21 by Alizarin Red and Oil Red O staining. Media were changed every 3–4 days, and staining was examined microscopically. **(A)** Osteogenic differentiation of ADSCs. **(B)** Adipogenic differentiation of ADSCs (Microscope ×100, Scale = 200 μm).

To evaluate the differentiation potential of ADSCs, the cells were subjected to osteogenic and adipogenic induction. By day 7 of osteogenic induction, minor cell overlap and a few calcified spots were observed, though staining intensity was not prominent. By day 14, the number of calcified spots increased, appearing orange upon staining, indicating the formation of calcified nodules. By day 21, Alizarin Red staining revealed numerous orange-red mineralized nodules, confirming successful differentiation of ADSCs into osteoblasts (Figure 2A). In the adipogenic induction, few translucent lipid droplets were visible near the nucleus by day 7. By day 14, lipid droplets increased in both number and size, and by day 21, substantial accumulation of orange-red lipid droplets of varying sizes was observed in the cytoplasm, indicating successful differentiation of ADSCs into adipocytes (Figure 2B).

### 3.3 Western blot detection of osteogenic and adipogenic differentiation-related proteins in rat ADSCs

**FIGURE 3:**
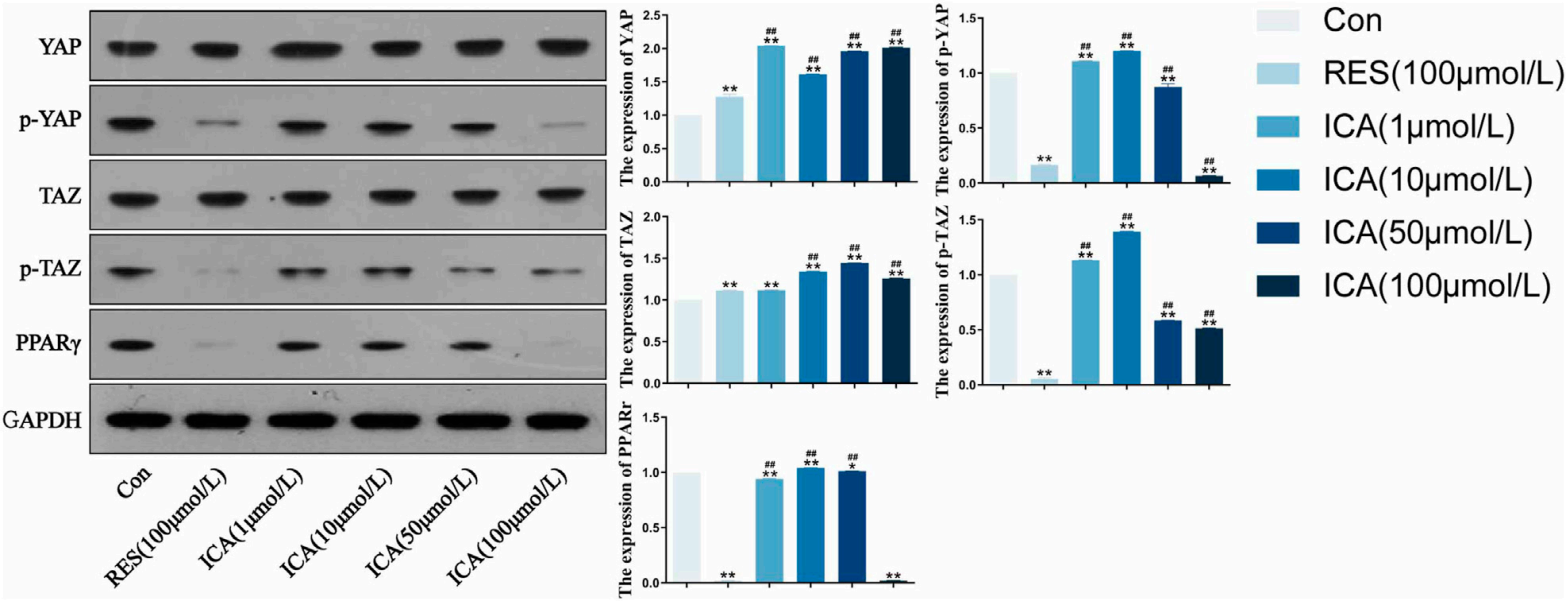
Western blot analysis of YAP, p-YAP, TAZ, p-TAZ and PPARγ expression in each group. ADSCs were subjected to Western blotting with the indicated antibodies. GAPDH was used as a control. Quantitative results of the relative protein levels are shown on the right side. The data were obtained from multiple analyses (n = 3). Statistical significance is indicated by: *p < 0.05, **p < 0.01 vs. the control group; #*p* < 0.05, ##*p* < 0.01 vs. the RES group.

Following treatment with different doses (1–100 μM) of ICA for 48 h, Western blot analysis assessed the expression levels of YAP, p-YAP, PPARγ, TAZ, and p-TAZ (Figure 3). The results demonstrated that, ICA treatment resulted in reduced phosphorylation levels of YAP and TAZ, enhanced their transcriptional activity, and inhibited the activity of the adipogenesis-related factor PPARγ, thereby promoting the osteogenic differentiation of ADSCs and suppressing adipogenic differentiation. Furthermore, a linear relationship was observed between YAP/TAZ/p-YAP/p-TAZ/PPARγ expression and ICA concentration.

### 3.4 Tracking detection of lentiviral infection of EGFP-ADSCs in rats

**FIGURE 4:**
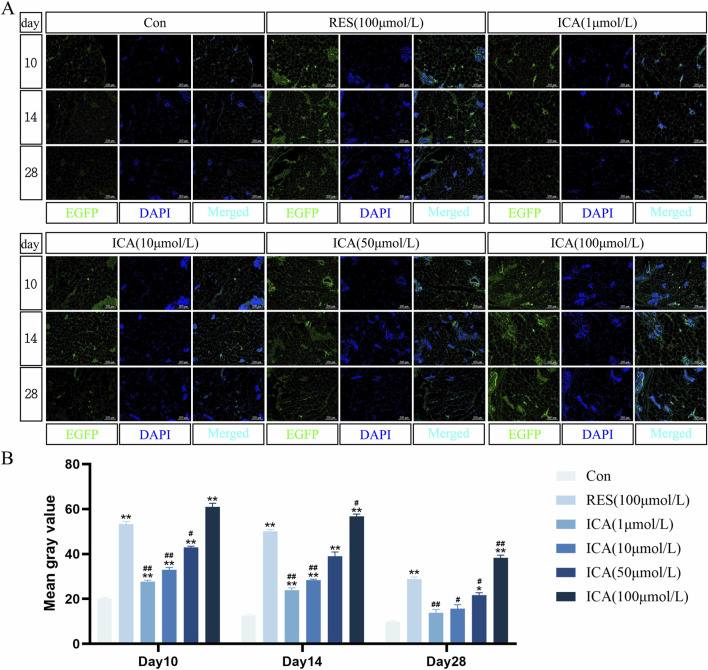
Tracing Detection of Lentiviral Infection of EGFP-ADSCs *In Vivo* in Rats. Rats were divided into six groups: control, resveratrol (100 μmol/L), and ICA (1, 10, 50, 100 μmol/L). Labeled ADSCs were injected subcutaneously, and tissue samples were collected at 10, 14, and 21 days post-injection for immunofluorescence analysis. **(A)** Immunofluorescent detection of EGFP-ADSCs in subcutaneous adipose tissue of rats (Microscope ×100, Scale = 200 μm). **(B)** Quantitative analysis of fluorescence intensity at different time points and in different groups of EGFP-ADSCs in subcutaneous adipose tissue of rats. The data were obtained from multiple analyses (n = 3). Statistical significance is indicated by: *p < 0.05, **p < 0.01 vs. the control group; #*p* < 0.05, ##*p* < 0.01 vs. the RES group.

On day 10 post-transplantation of ADSCs, fluorescence was observed in the blank group, control group, and ICA-treated groups (1, 10, 50, 100 μmol/L). The control and 100 μmol/L ICA groups exhibited stronger fluorescence, whereas the blank group and lower ICA concentrations showed weaker fluorescence. By days 14 and 28, fluorescence intensity gradually decreased in all groups, with near undetectable levels in the blank group and the 1 μmol/L ICA group by day 28 (Figure 4A). Quantitative analysis of fluorescence intensity at different time points and in different groups of EGFP-ADSCs in subcutaneous adipose tissue of rats (Figure 4B).

### 3.5 Immunohistochemical detection of the expression of genes related to proliferation, osteogenesis, and adipogenesis

#### 3.5.1 Proliferation-related gene BrdU

**FIGURE 5:**
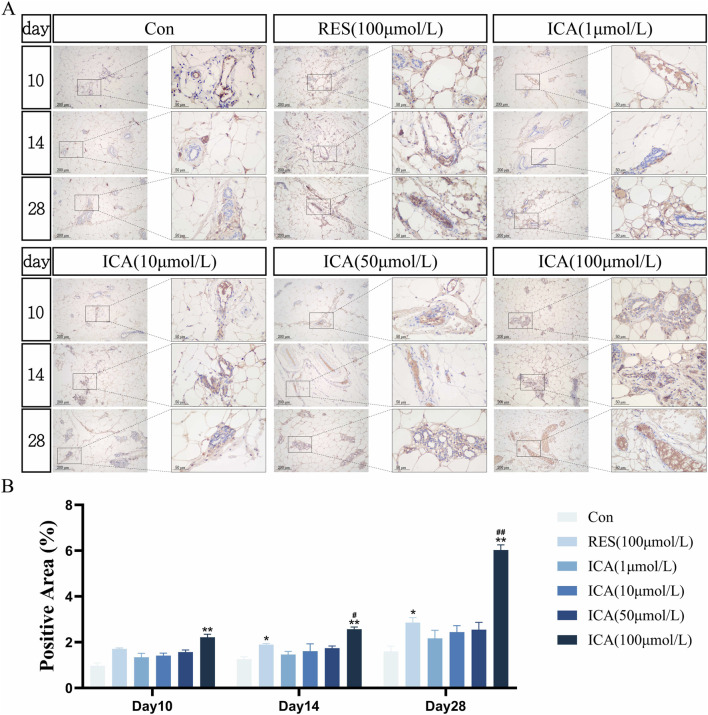
Immunohistochemical Detection of the Expression of Genes Related to Proliferation. Rats were divided into six groups: control, resveratrol (100 μmol/L), and ICA (1, 10, 50, 100 μmol/L). Tissue samples were collected at 10, 14, and 21 days for immunohistochemical analysis. **(A)** Expression of the proliferation-related gene BrdU detected by immunohistochemistry (Left: Microscope ×100, Scale = 200 μm; Right: Microscope ×400, Scale = 50 μm). **(B)** Quantitative analysis of the BrdU-positive cell area. The data were obtained from multiple analyses (n = 3). Statistical significance is indicated by: *p < 0.05, **p < 0.01 vs. the control group; #*p* < 0.05, ##*p* < 0.01 vs. the RES group.

On day 10 post-transplantation, numerous BrdU-positive nuclei, indicative of cell proliferation, were observed at the transplantation sites in the ADSC, RES, and ICA-treated groups (1, 10, 50, and 100 μmol/L). The number of positive cells increased in all groups over time, with 100 μmol/L ICA groups exhibiting the highest counts of positive cells (Figure 5A). Quantitative analysis of the BrdU-positive cell area at different time points and in various groups within the subcutaneous adipose tissue of rats (Figure 5B).

#### 3.5.2 Osteogenesis-related gene ALP

**FIGURE 6:**
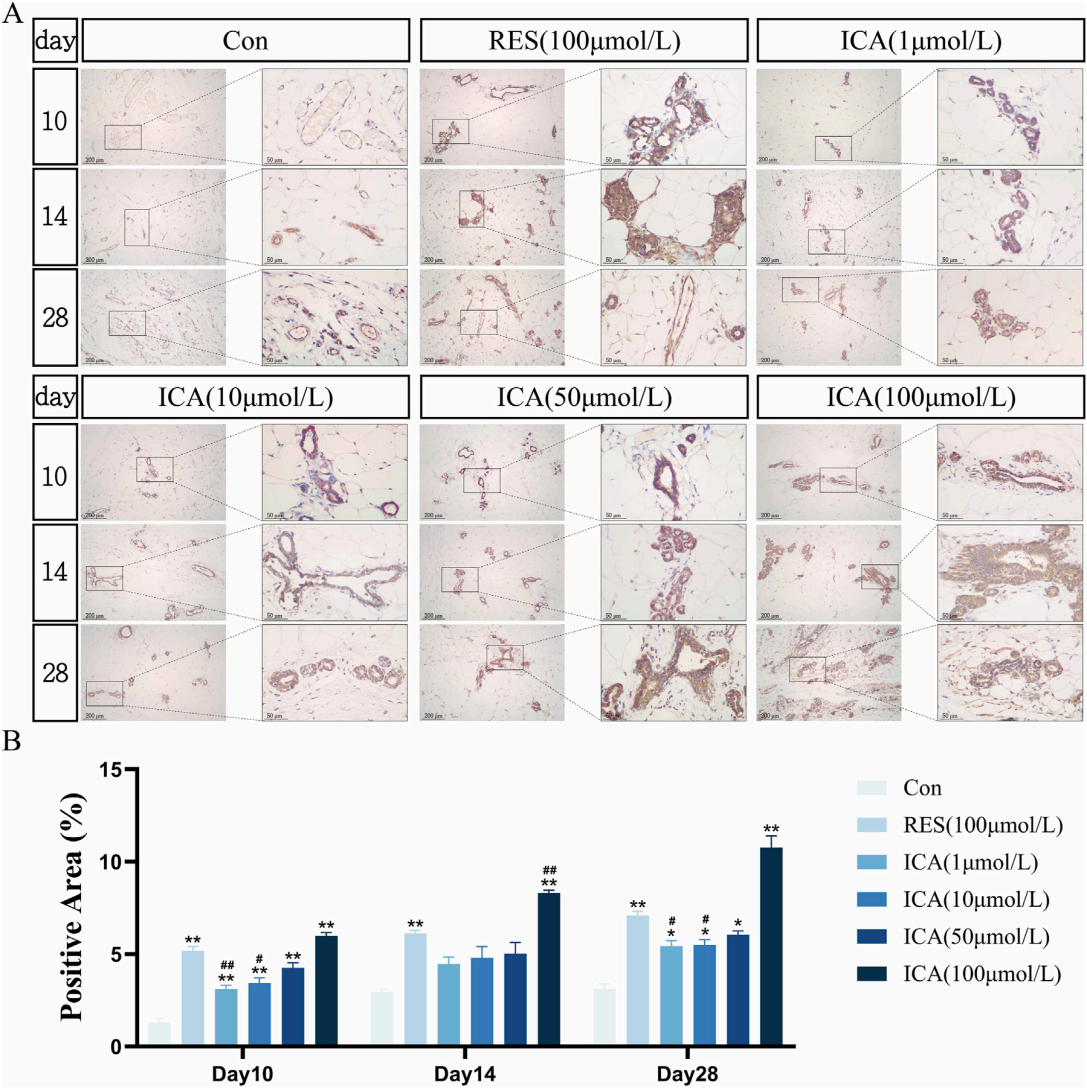
Immunohistochemical Detection of the Expression of Genes Related to Osteogenesis. Rats were divided into six groups: control, resveratrol (100 μmol/L), and ICA (1, 10, 50, 100 μmol/L). Tissue samples were collected at 10, 14, and 21 days for immunohistochemical analysis. **(A)** Expression of the osteogenesis-related gene ALP detected by immunohistochemistry (Left: Microscope ×100, Scale = 200 μm; Right: Microscope ×400, Scale = 50 μm). **(B)** Quantitative analysis of the ALP-positive cell area. The data were obtained from multiple analyses (n = 3). Statistical significance is indicated by: *p < 0.05, **p < 0.01 vs. the control group; #*p* < 0.05, ##*p* < 0.01 vs. the RES group.

On day 10 post-transplantation, numerous ALP-positive cells related to osteogenesis were observed in the ADSC, RES, and ICA-treated groups (1, 10, 50, and 100 μmol/L). The number of ALP-positive cells increased over time across all groups, with 100 μmol/L ICA groups showing the highest counts (Figure 6A). Quantitative analysis of the ALP-positive cell area at different time points and in various groups within the subcutaneous adipose tissue of rats (Figure 6B).

#### 3.5.3 Osteogenesis-related gene Stro-1

**FIGURE 7:**
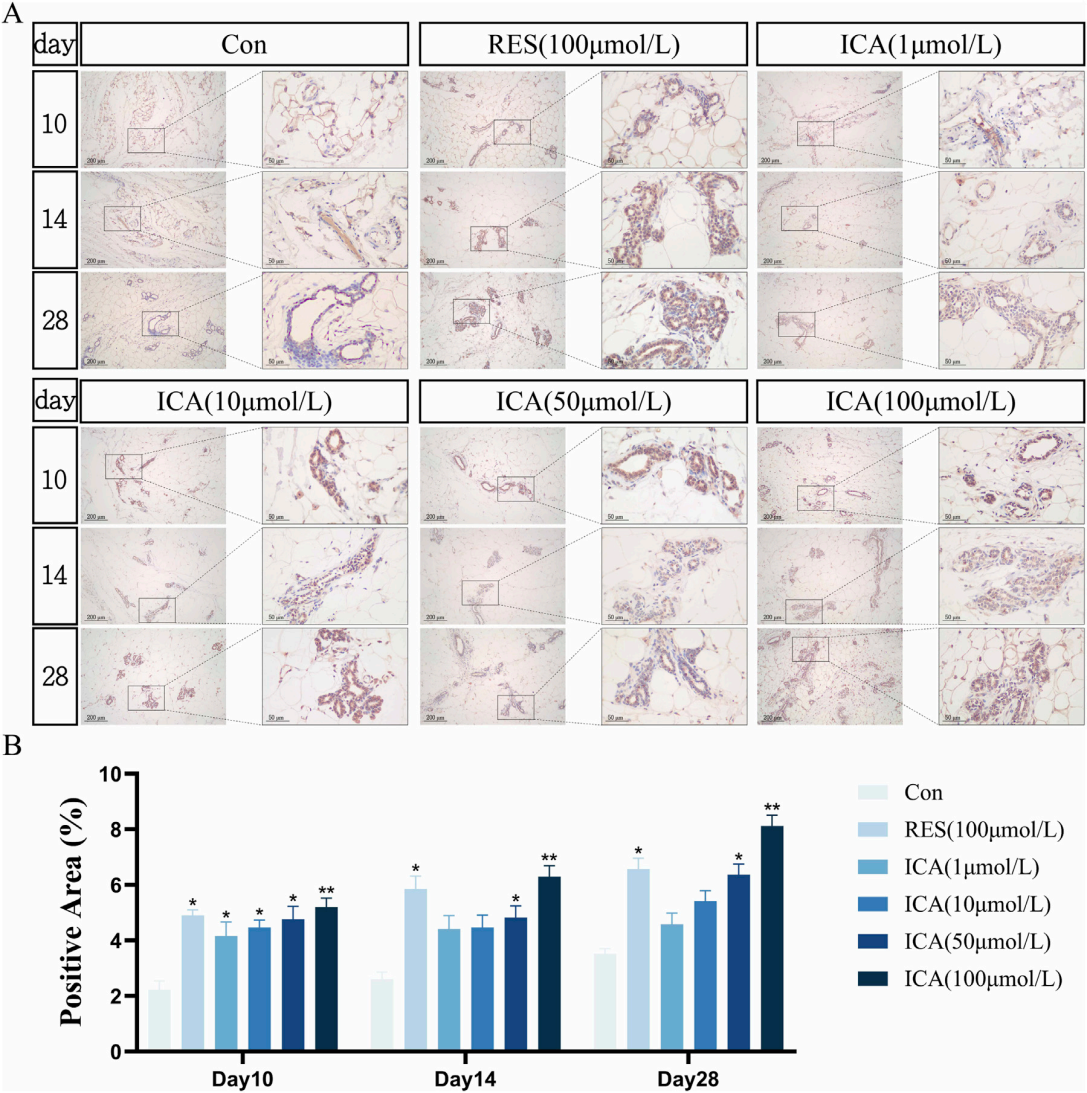
Immunohistochemical Detection of the Expression of Genes Related to Osteogenesis. Rats were divided into six groups: control, resveratrol (100 μmol/L), and ICA (1, 10, 50, 100 μmol/L). Tissue samples were collected at 10, 14, and 21 days for immunohistochemical analysis. **(A)** Expression of the osteogenesis-related gene Stro-1 detected by immunohistochemistry (Left: Microscope ×100, Scale = 200 μm; Right: Microscope ×400, Scale = 50 μm). **(B)** Quantitative analysis of the Stro-1-positive cell area. The data were obtained from multiple analyses (n = 3). Statistical significance is indicated by: *p < 0.05, **p < 0.01 vs. the control group; #*p* < 0.05, ##*p* < 0.01 vs. the RES group.

On day 10 post-transplantation, a significant number of Stro-1-positive cells related to osteogenesis were observed at the transplantation sites in the ADSC, RES, and ICA-treated groups (1, 10, 50, and 100 μmol/L). The number of positive cells increased across all groups over time, with 100 μmol/L ICA groups exhibiting the highest counts (Figure 7A). Quantitative analysis of the Stro-1-positive cell area at different time points and in various groups within the subcutaneous adipose tissue of rats (Figure 7B).

#### 3.5.4 Osteogenesis-related gene PGC-1α

**FIGURE 8:**
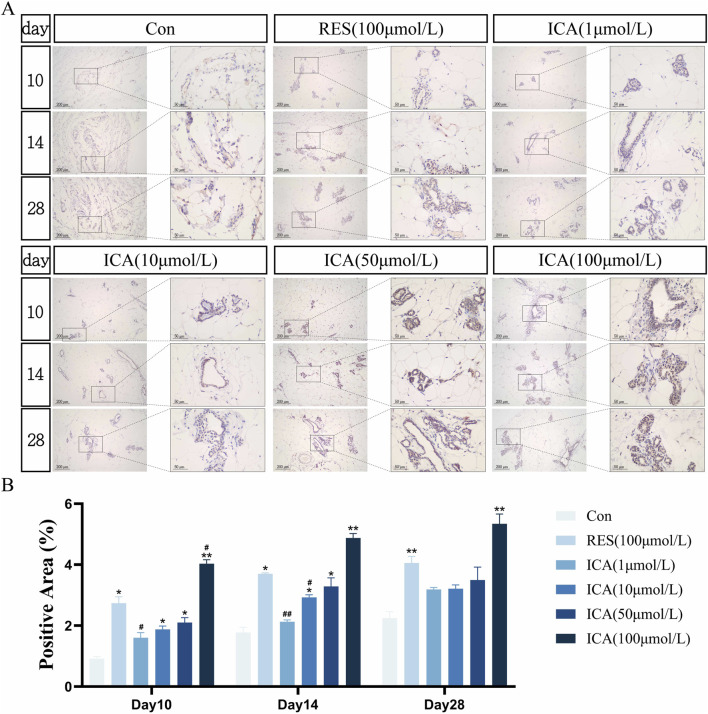
Immunohistochemical Detection of the Expression of Genes Related to Osteogenesis. Rats were divided into six groups: control, resveratrol (100 μmol/L), and ICA (1, 10, 50, 100 μmol/L). Tissue samples were collected at 10, 14, and 21 days for immunohistochemical analysis. **(A)** Expression of the osteogenesis-related gene PGC-1α detected by immunohistochemistry (Left: Microscope ×100, Scale = 200 μm; Right: Microscope ×400, Scale = 50 μm). **(B)** Quantitative analysis of the PGC-1α-positive cell area. The data were obtained from multiple analyses (n = 3). Statistical significance is indicated by: *p < 0.05, **p < 0.01 vs. the control group; #*p* < 0.05, ##*p* < 0.01 vs. the RES group.

On day 10 post-transplantation, a significant number of PGC-1α-positive cells associated with osteogenesis were observed in the ADSC, RES, and ICA-treated groups (1, 10, 50, and 100 μmol/L). The number of positive cells increased over time across all groups, with 100 μmol/L ICA groups exhibiting the highest counts (Figure 8A). Quantitative analysis of the PGC-1α-positive cell area at different time points and in various groups within the subcutaneous adipose tissue of rats (Figure 8B).

#### 3.5.5 Adipogenesis-related gene PPARγ

**FIGURE 9:**
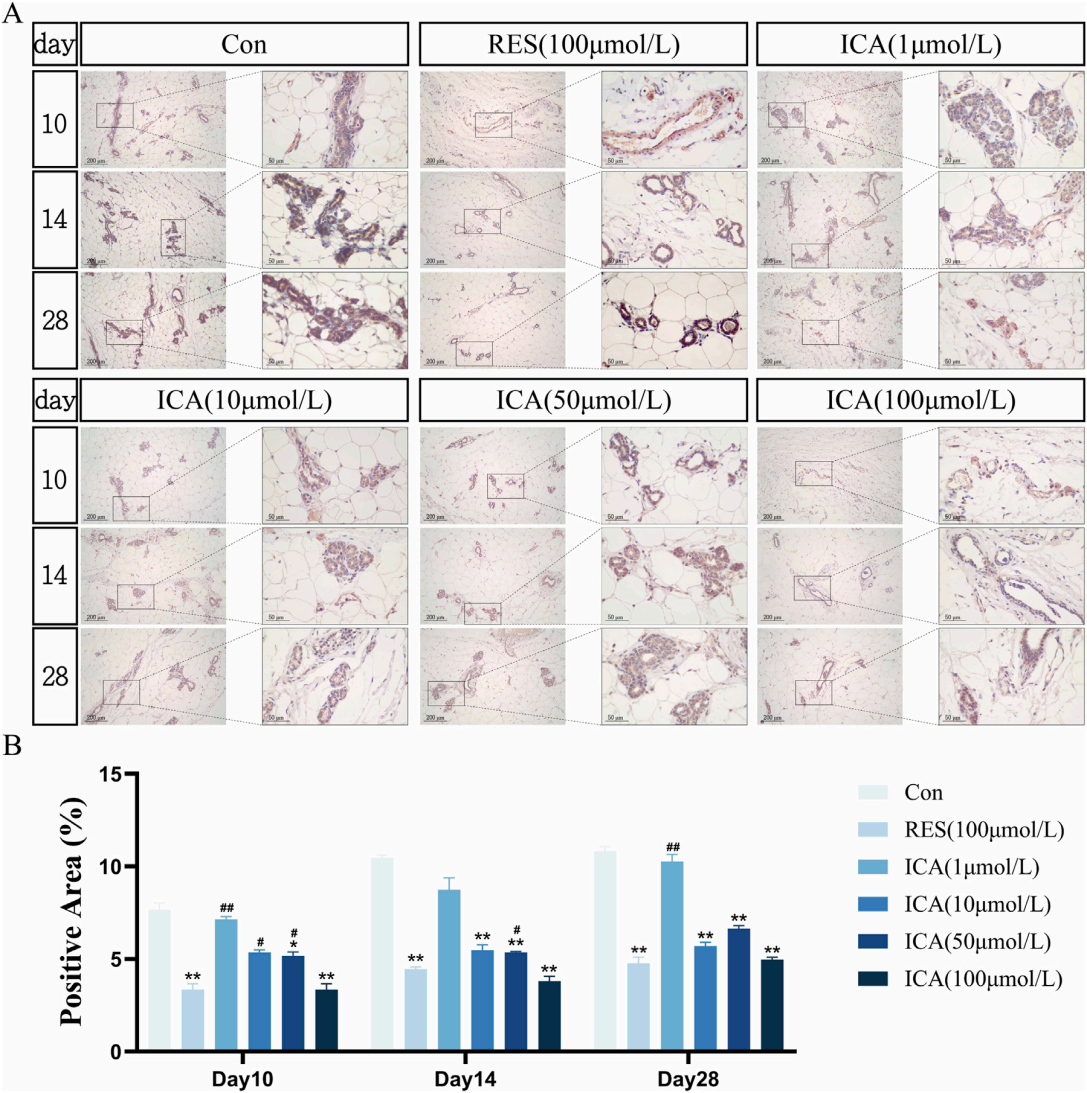
Immunohistochemical Detection of the Expression of Genes Related to Adipogenesis. Rats were divided into six groups: control, resveratrol (100 μmol/L), and ICA (1, 10, 50, 100 μmol/L). Tissue samples were collected at 10, 14, and 21 days for immunohistochemical analysis. **(A)** Expression of the adipogenesis-related gene PPARγ detected by immunohistochemistry (Left: Microscope ×100, Scale = 200 μm; Right: Microscope ×400, Scale = 50 μm). **(B)** Quantitative analysis of the PPARγ-positive cell area. The data were obtained from multiple analyses (n = 3). Statistical significance is indicated by: *p < 0.05, **p < 0.01 vs. the control group; #*p* < 0.05, ##*p* < 0.01 vs. the RES group.

On day 10 post-transplantation, PPARγ-positive cells associated with adipogenesis were observed in the ADSC, RES, and ICA-treated groups. Positive cell numbers increased over time, with higher counts in the ADSC and lower concentration ICA groups, while 100 μmol/L ICA groups exhibited relatively fewer positive cells (Figure 9A). Quantitative analysis of the PPARγ-positive cell area at different time points and in various groups within the subcutaneous adipose tissue of rats (Figure 9B).

## 4 Discussion

Osteoporosis is a long-term condition that leads to reduced bone density and affects millions of people worldwide. The limited ability of bones to regenerate makes repairing bone damage a major challenge in orthopedic and regenerative medicine. Although bone mesenchymal stem cells (BMSCs) are well-studied for their ability to self-renew and differentiate into multiple cell types, their extraction is invasive and supplies are limited, making them unsuitable for widespread clinical use in advanced disease stages (Guo et al., 2022; Mohamed-Ahmed et al., 2021; Zhu et al., 2021). In contrast, adipose-derived stem cells (ADSCs) offer several benefits: they are easy to obtain, straightforward to isolate, can be extensively expanded *in vitro*, and have the potential for cross-germ layer differentiation. These qualities position ADSCs as promising candidates for research and treatment of osteoporosis.

Several traditional Chinese medicine (TCM) compounds, such as Zuogui pill, Yougui pill, puerarin, salidroside, and total glycosides from Eucommia ulmoides seeds, have been reported to induce osteogenic differentiation in ADSCs (Li et al., 2018; Li et al., 2021; Wang et al., 2011; Zhou and Xie, 2021). Epimedium has been widely used in traditional Chinese medicine for treating osteoporosis. Clinical studies have shown that Epimedium-derived phytoestrogen flavonoids effectively prevent bone loss in late postmenopausal women by maintaining bone mineral density and reducing bone resorption markers without adverse effects on serum estradiol or endometrial thickness (Zhang et al., 2007). Additionally, Epimedium prenylflavonoids are safe for short-term use, increase serum levels of bone anabolic markers such as bone-specific alkaline phosphatase, and may reduce osteoclast activity through suppression of TRAF6 in precursor monocytes. These findings support its potential as a therapeutic agent for osteoporosis by demonstrating both safety and efficacy in enhancing bone health and preventing bone loss (Yong et al., 2021). Building on this evidence, this study focuses on Icariin (ICA), the bioactive component of Epimedium, to explore its effects on the osteogenic differentiation of ADSCs *in vitro* and elucidate the underlying mechanisms promoting bone formation.

The Hippo signaling pathway is crucial for controlling organ size, wound healing, and tissue regeneration, and it significantly impacts osteogenesis (Moya and Halder, 2019). Yes-associated protein (YAP) and transcriptional coactivator with PDZ-binding motif (TAZ) are key downstream coactivators of the Hippo pathway and are known to promote osteogenesis (Kovar et al., 2020). YAP and TAZ regulate skeletal development by modulating osteoblast activity, osteoclast-mediated remodeling, and matrix composition (Kegelman et al., 2018). Specifically, TAZ enhances osteogenic differentiation as a coactivator of RUNX2, a key transcription factor that upregulates osteogenesis-related genes (Xue et al., 2013). The presence of TAZ in the nucleus is essential for its interaction with transcription factors and the activation of target genes. The localization and activity of TAZ are influenced by external signals. These signals include the Hippo and Wnt pathways (Lei et al., 2008; Liu et al., 2010). Additionally, TAZ suppresses adipogenesis by interacting with peroxisome proliferator-activated receptor γ (PPARγ) (Cui et al., 2003; Hong et al., 2005). Notably, The TAZ agonist TM-25659 notably increases TAZ expression in adipose-derived stem cells (ADSCs), which enhances their ability to differentiate into bone-forming cells *in vivo*. This highlights TAZ as a vital regulator of osteogenic differentiation in ADSCs. It also suggests that targeting TAZ pharmacologically could effectively guide stem cell differentiation, promote bone regeneration and repair, and reduce bone loss (Jang et al., 2012). Likewise, YAP has comparable effects in bone marrow-derived mesenchymal stem cells (BMSCs). Phosphorylated YAP interacts with RUNX2 and PPARγ in the nucleus. This interaction promotes osteogenic differentiation and inhibits adipogenic differentiation. Additionally, there is a positive correlation between the level of osteogenic differentiation and cytoskeletal density, emphasizing the relationship between cytoskeletal organization and osteogenic activity (Lei et al., 2008).

Our study found that ICA significantly increased the expression of YAP and TAZ while decreasing the levels of phosphorylated YAP and TAZ in a dose-dependent manner. These findings indicate that ICA promotes the osteogenic differentiation of ADSCs by inhibiting the Hippo pathway. This inhibition reduces YAP and TAZ phosphorylation and facilitates their translocation into the nucleus. Furthermore, we observed a linear relationship between the expression levels of YAP and TAZ and the concentration of ICA.

PPARγ is recognized as a master regulator of adipogenesis, and no other factors are known to trigger this process without its presence (Stachecka et al., 2019). PPARγ, a ligand-activated nuclear hormone receptor, plays a crucial role in initiating adipocyte differentiation. It also promotes the expression of lipogenic genes, which contributes to lipid accumulation (Lefterova et al., 2014). Both human and animal studies have shown an increase in PPARγ expression during the differentiation of fat cells (Lee et al., 2019). Our findings show that ICA at a concentration of 100 μmol/L significantly reduces PPARγ expression. This suggests that ICA inhibits this adipogenic factor during the osteogenesis of ADSCs, thereby promoting their differentiation into bone-forming cells.

When the Hippo signaling pathway is activated, YAP/TAZ undergo phosphorylation, are sequestered in the cytoplasm, or are degraded through the ubiquitination pathway, leading to inhibited cell proliferation. Conversely, when the Hippo pathway is inhibited, dephosphorylated YAP/TAZ move to the nucleus and interact with transcription factors TEAD1-4, promoting the expression of genes related to proliferation and migration (Chen et al., 2019; Kovar et al., 2020; Xiong et al., 2018). The movement of YAP/TAZ between the cytoplasm and nucleus, based on their phosphorylation status, regulates the physiological activities of osteoblasts, osteoclasts, and chondrocytes. (Xiong et al., 2018).

While YAP/TAZ directly inhibits PPARγ, potential crosstalk with Wnt/β-catenin or AMPK pathways cannot be excluded. Our findings show that ICA at a concentration of 100 μmol/L promotes osteogenic differentiation in ADSCs. This occurs through the inhibition of PPARγ expression and the reduction of YAP/TAZ phosphorylation, leading to enhanced osteogenesis and decreased adipogenesis. These molecular mechanisms synergistically enhance osteogenesis while suppressing adipogenesis, thereby shifting the differentiation balance toward bone formation. This dual regulatory role highlights ICA as a promising pharmacological candidate for developing treatments aimed at bone regeneration and metabolic bone disorders.

Although most studies on ADSC osteogenic differentiation have been conducted *in vitro*, the *in vivo* survival, proliferation, distribution, and differentiation of ADSCs after transplantation are still not well understood. The survival and proliferation of ADSCs after transplantation are essential for effective tissue repair. A significant challenge in stem cell therapy is the high rate of cell death, including apoptosis and necrosis, that occurs when stem cells are transplanted into regions with poor blood flow and low oxygen levels, such as the infarcted myocardium. Previous research has shown that ICA can reduce apoptosis in H9c2 rat cardiomyocytes by blocking the JNK/NF-κB signaling pathway influenced by reactive oxygen species (Zhou et al., 2015). In our *in vivo* study, we observed that ICA-induced bone formation was noticeable by day 10 and continued until day 28. Similarly, the suppression of fat cell formation was consistent, indicating enduring long-term effects. These findings show that ICA-treated ADSCs were effectively distributed and survived after subcutaneous injection into allogeneic rats. Furthermore, the ICA-treated ADSCs demonstrated increased bone formation and a reduction in fat cell formation.

## 5 Conclusion

In conclusion, our study shows that ICA-treated ADSCs successfully localize and survive at the injection site following subcutaneous transplantation in allogeneic rats, promoting osteogenic differentiation and inhibiting adipogenesis. This effect likely results from the inhibition of the Hippo signaling pathway, which reduces YAP/TAZ phosphorylation, prevents YAP/TAZ from translocating to the nucleus, and suppresses the adipogenic regulator PPARγ. While additional research is required to reveal the exact mechanisms of YAP/TAZ regulation in the cytoplasm and nucleus, our findings significantly advance our understanding of how ICA influences ADSC differentiation. Furthermore, ICA’s ability to modulate both osteogenesis and adipogenesis makes it a promising treatment for osteoporosis, metabolic bone diseases, and bone defects, offering a comprehensive approach to restoring skeletal health. These results provide critical experimental evidence supporting the clinical application of ADSCs for bone-related therapies, offering a comprehensive strategy to improve skeletal health.

## Data Availability

The original contributions presented in the study are included in the article/supplementary material, further inquiries can be directed to the corresponding authors.
